# Exploiting natural regulation

**DOI:** 10.7554/eLife.39664

**Published:** 2018-08-06

**Authors:** Catherine Proenza

**Affiliations:** 1 Department of Physiology and BiophysicsUniversity of Colorado Anschutz Medical CampusAuroraUnited States; 2 Department of Medicine, Division of CardiologyUniversity of Colorado Anschutz Medical CampusAuroraUnited States

**Keywords:** cardiac pacemaker, HCN channels, cAMP, TRIP8b, allosteric regulation, Mouse

## Abstract

Using a short peptide to regulate the activity of HCN ion channels illustrates how physiological modulators could inspire new drugs.

**Related research article** Saponaro A, Cantini F, Porro A, Bucchi A, DiFrancesco D, Maione V, Donadoni C, Introini B, Mesirca P, Mangoni ME, Thiel G, Banci L, Santoro B, Moroni A. 2018. A synthetic peptide that prevents cAMP regulation in mammalian hyperpolarization-activated cyclic nucleotide-gated (HCN) channels. *eLife* **7**:e35753. doi: 10.7554/eLife.35753

From the brain to the heart to the bladder, electrical potentials across cell membranes ensure that nerves and muscles work properly. These potentials emerge as ions go into and out of cells, crossing the membrane through proteins known as ion channels. The structures open and close their ion-conducting pores in response to voltage changes, the binding of ligands, or both.

Mutations in ion channels can lead to conditions like epilepsy or abnormal cardiac rhythms, which are caused by faulty electric signals in the brain or the heart. Drugs that target these proteins can help to treat the diseases, but they often act by blocking the pore. This means that they can inhibit but not increase the activity of the channel. Moreover, given the similarities between the pores of different types of ion channels, it is challenging to create blockers that are specific to just one type.

Nature regulates ion channels too, of course. For example, our heart beats faster during a fight-or-flight response because the body subtly alters the properties of ion channels in the pacemaker cells that set the heart rate. However, nature does not typically block pores: rather, it relies on molecules that act indirectly, via other domains of the channels. This ‘allosteric modulation’ can either activate or inhibit ion channels; it can also be more specific than pore blockade because these other domains vary considerably between different types of channels. Designing new categories of drugs with sites of action that mimic the ones found in natural allosteric regulators could help to target ion channels more accurately, and control their activity more finely.

Now, in eLife, Anna Moroni of the University of Milan, Bina Santoro at Columbia University, and colleagues at institutes in Italy, Germany and France – including Andrea Saponaro as first author – report what may constitute a first step towards developing such drugs (Saponaro et al., 2018).

Their work focuses on HCN (short for hyperpolarization-activated cyclic nucleotide-sensitive) channels, which are present both in neurons and in cardiac pacemaker cells. These channels open when the membrane potential becomes more negative, but nature also uses cyclic nucleotides such as cAMP to fine-tune the precise voltage range at which they activate. Cyclic nucleotides attach directly to a conserved sequence, the cyclic nucleotide-binding domain, which is located in an intracellular region of HCN channels; in turn, this interaction shifts the range of voltage activation to more positive potentials, making the channels easier to open with voltage.

Nearly fifteen years ago, it was found that HCN channels in neurons associate with an additional subunit called TRIP8b (Santoro et al., 2004). This protein binds to the channels at two distinct sites, which in turn modulates their activity in two different ways (Santoro et al., 2011; Han et al., 2011; Bankston et al., 2012). In one type of regulation, an end of TRIP8b associates with one of the extremities of the channels to control how many channels are expressed on the membrane. In the other type, the central core region of TRIP8b attaches to the cyclic nucleotide-binding domain to decrease the sensitivity of the channel to cAMP.

Building on previous research, Saponaro et al. characterize the interactions between TRIP8b and HCN channels that are required to lessen their response to cAMP. Using a combination of techniques (NMR, structural modeling, and calorimetry) they reveal that a short peptide containing 40 amino acids, TRIP8b_nano_, attaches to the cyclic nucleotide-binding domain and is enough to reduce the affinity of the channel for cAMP.

The interactions between TRIP8b_nano_ and the cyclic nucleotide-binding domain were found to be mainly electrostatic, as previously suggested (DeBerg et al., 2015). Many, but not all, of the residues that associate with TRIP8b_nano_ also interact with cAMP. This observation supports previous claims that the channels reduce their affinity for cAMP in two ways. First, TRIP8b and the nucleotide directly compete with each other (Han et al., 2011; DeBerg et al., 2015; Bankston et al., 2017); second, when TRIP8b binds, it indirectly causes rearrangements in the cyclic nucleotide-binding domain that make it more difficult for cAMP to attach (Hu et al., 2013; Saponaro et al., 2014; DeBerg et al., 2015).

An important set of experiments also showed that the TRIP8b_nano_ peptide can prevent cAMP from regulating HCN channels which are both native (in cardiac pacemaker cells) and artificially expressed (in propagated cell lines). In fact, even though TRIP8b is not expressed in the heart, the peptide can still slow the rate at which pacemaker cells fire; in principle, this could be used to design better drugs to decrease heart rate. As the interaction between HCN channels and TRIP8b has also been associated with depression (Lyman et al., 2017) and active coping behavior (Fisher et al., 2018), these results may be of interest in the treatment of mental health disorders as well.

The work by Saponaro et al. is a proof of concept that small regions of natural allosteric regulators can be exploited to modify the activity of ion channels in cells. Ultimately, it underscores how pinpointing regulatory hotspots on these channels could power the creation of new allosteric drugs.

## References

[bib1] Bankston JR, Camp SS, DiMaio F, Lewis AS, Chetkovich DM, Zagotta WN (2012). Structure and stoichiometry of an accessory subunit TRIP8b interaction with hyperpolarization-activated cyclic nucleotide-gated channels. PNAS.

[bib2] Bankston JR, DeBerg HA, Stoll S, Zagotta WN (2017). Mechanism for the inhibition of the cAMP dependence of HCN ion channels by the auxiliary subunit TRIP8b. Journal of Biological Chemistry.

[bib3] DeBerg HA, Bankston JR, Rosenbaum JC, Brzovic PS, Zagotta WN, Stoll S (2015). Structural mechanism for the regulation of HCN ion channels by the accessory protein TRIP8b. Structure.

[bib4] Fisher DW, Han Y, Lyman KA, Heuermann RJ, Bean LA, Ybarra N, Foote KM, Dong H, Nicholson DA, Chetkovich DM (2018). HCN channels in the hippocampus regulate active coping behavior. Journal of Neurochemistry.

[bib5] Han Y, Noam Y, Lewis AS, Gallagher JJ, Wadman WJ, Baram TZ, Chetkovich DM (2011). Trafficking and gating of hyperpolarization-activated cyclic nucleotide-gated channels are regulated by interaction with tetratricopeptide repeat-containing Rab8b-interacting protein (TRIP8b) and cyclic AMP at distinct sites. Journal of Biological Chemistry.

[bib6] Hu L, Santoro B, Saponaro A, Liu H, Moroni A, Siegelbaum S (2013). Binding of the auxiliary subunit TRIP8b to HCN channels shifts the mode of action of cAMP. The Journal of General Physiology.

[bib7] Lyman KA, Han Y, Chetkovich DM (2017). Animal models suggest the TRIP8b-HCN interaction is a therapeutic target for major depressive disorder. Expert Opinion on Therapeutic Targets.

[bib8] Santoro B, Hu L, Liu H, Saponaro A, Pian P, Piskorowski RA, Moroni A, Siegelbaum SA (2011). TRIP8b regulates HCN1 channel trafficking and gating through two distinct C-terminal interaction sites. Journal of Neuroscience.

[bib9] Santoro B, Wainger BJ, Siegelbaum SA (2004). Regulation of HCN channel surface expression by a novel C-terminal protein-protein interaction. Journal of Neuroscience.

[bib10] Saponaro A, Cantini F, Porro A, Bucchi A, DiFrancesco D, Maione V, Donadoni C, Introini B, Mesirca P, Mangoni ME, Thiel G, Banci L, Santoro B, Moroni A (2018). A synthetic peptide that prevents cAMP regulation in mammalian hyperpolarization-activated cyclic nucleotide-gated (HCN) channels. eLife.

[bib11] Saponaro A, Pauleta SR, Cantini F, Matzapetakis M, Hammann C, Donadoni C, Hu L, Thiel G, Banci L, Santoro B, Moroni A (2014). Structural basis for the mutual antagonism of cAMP and TRIP8b in regulating HCN channel function. PNAS.

